# Prediction of potential habitat of *Isodon amethystoides* in China under climate change based on optimized MaxEnt model

**DOI:** 10.3389/fpls.2025.1657417

**Published:** 2025-09-10

**Authors:** Qiya Ji, Shimao Chen, Yunan Cao, Xiaosu Xiang, Yunyun Sun, Yan Zhang, Wanbo Xing, Yanyan Wang, Qingshan Yang

**Affiliations:** ^1^ College of Pharmacy, Anhui University of Chinese Medicine, Hefei, China; ^2^ Anhui Key Laboratory of Traditional Chinese Medicine Research and Development, Hefei, China; ^3^ Institute of Conservation and Development of Traditional Chinese Medicine Resources, Anhui Academy of Chinese Medicine, Hefei, China

**Keywords:** *Isodon amethystoides*, MaxEnt model, environmental variables, climate change, habitat distribution

## Abstract

*Isodon amethystoides* (Benth.) H. Hara, a plant species belonging to the genus Isodon in the Lamiaceae family, possesses multiple medicinal properties including heat-clearing and detoxifying effects, anti-inflammatory and antibacterial activities, as well as liver-protective functions. Due to the challenges in harvesting from wild sources, current production primarily relies on artificial cultivation. Compared with wild resources, artificial cultivation not only improves medicinal material quality through standardized planting practices, but also enhances the content of bioactive components. Furthermore, it enables scientific evaluation of environmental factors’ impact on medicinal quality. This study uses the Maxent model to predict the current and future potential distribution areas and suitable habitats for Isodon amethystoides. Based on 194 species occurrence records and 90 environmental variables, we identified key environmental factors influencing its distribution through correlation analysis and variable contribution assessment, followed by model parameter optimization. The optimized model achieved an AUC value of 0.902, demonstrating excellent predictive performance. The results demonstrated that under current climatic conditions, the total suitable habitat area for *Isodon amethystoides* was estimated at 2.08×10^6^ km², accounting for 21.66% of China’s terrestrial land area. The key environmental factors affecting the distribution of potential suitable habitats for *Isodon amethystoides* are precipitation in September, monthly precipitation, and standard deviation of seasonal temperature variation. Under future climate change scenarios (SSP1-2.6 and SSP5-8.5), the total suitable habitat area of *Isodon amethystoides* shows an overall increasing trend. By the 2050s, the suitable area is projected to reach its maximum extent approximately 2.48×10^6^ km², with primary expansion occurring toward the northwest. Notably, Yunnan Province exhibits significant habitat expansion, while the centroid of suitable habitat consistently remains located in Chongqing. This study provides scientific support for the conservation of wild *Isodon amethystoides* resources and the planning of cultivation areas, thereby contributing to sustainable development and ecological protection.

## 1 Introduction

Climate is a crucial factor influencing species changes. Global warming has caused significant alterations in temperature and precipitation patterns, a trend that exerts severe impacts on plant growth environments (Li et al., 2019; Mishra, 2021). Meanwhile, the frequent occurrence of extreme weather events and changes in precipitation patterns have directly led to a decrease in the yield of medicinal plants in China. This has also put some species at risk of extinction due to their inability to adapt to the rapidly changing climatic environment (Barnosky and Wiens, 2016). To address this situation, researchers have developed various ecological modeling methods in recent years, mainly including Ecological Niche Factor Analysis (ENFA), Bioclimatic Analysis System (BIOCLIM), Genetic Algorithm for Rule-set Production (GARP), and Maxent model (Phillips et al., 2006). Among them, the Maximum Entropy Model (MaxEnt) is widely used due to its high accuracy, low requirement for sample size, and stable and reliable operation results. For example, MaxEnt has been widely applied to the suitable area zoning of medicinal plants such as *Atractylodes lancea*, *Verbena officinalis*, and *Cirsium lineare*, providing important scientific basis for the protection, cultivation, and sustainable development of medicinal plant resources (Fang et al., 2024; Chen et al., 2025).

*Isodon amethystoides* is a herbaceous plant in the family Lamiaceae, widely distributed across China, is primarily distributed in subtropical montane regions between 23°N and 34°N in China. Its natural populations predominantly occur at elevations of 300-1,500 m, inhabiting forest margins and shrublands under warm-humid climatic conditions The species shows strong edaphic preference for acidic soils (red/yellow podzolic soils, pH 4.5-6.0) and typically forms symbiotic communities with Ericaceae (e.g.,*Rhododendron* spp.) and Fagaceae (e.g., *Castanopsis* spp.) species. *Isodon amethystoides* uses its stems and leaves as medicinal parts, possessing the effects of clearing heat and detoxifying, promoting blood circulation and removing blood stasis, anti-inflammation and analgesia. It is commonly used in the treatment of hepatitis, gastritis, tumors and other diseases (Duan et al., 2021; Zhao et al., 2022; Zhou et al., 2024). *Isodon amethystoides* also has complex and diverse chemical compositions, including diterpenoids, triterpenoids, flavonoids, phenolic acids, and volatile oils (Jin et al., 2010). Among them, diterpenoids are the characteristic components and the material basis for anti-tumor and antibacterial activities; flavonoids and phenolic acids are mainly used for anti-inflammatory and antioxidant effects. In recent years, new compounds have been continuously discovered in the genus *Isodon*, providing a scientific basis for the development of its medicinal value (Jiang et al., 2021). As a traditional Chinese medicinal material, the market supply of *Isodon amethystoides* mainly relies on artificial cultivation. However, artificial cultivation is constrained by problems such as poor environmental adaptability and scarcity of high-quality germplasm, making it difficult to meet the growing market demand. Therefore, using the MaxEnt model to deeply study the key environmental factors affecting the suitable growth of *Isodon amethystoides* and accurately predict the distribution of its potential suitable areas can provide theoretical guidance for the site selection and standardized cultivation of artificial cultivation.

This study takes China as the research scope, collects and collates distribution information based on field surveys and previous specimen records, combines environmental factors such as climate, topography, and soil, sets the optimal parameters of the MaxEnt model through analysis using the Kuenm package, simulates and predicts the distribution and changes of *Isodon amethystoides* habitats under the current and future climate change contexts. The adoption of the Maxent model for predicting the suitable habitats of *Isodon amethystoides* effectively addresses the challenge posed by limited distribution data for this medicinal plant.With the aid of ArcGIS, it analyzes and displays the size of suitable distribution areas and the change patterns of distribution centroids, providing theoretical references for the scientific protection and rational development of wild *Isodon amethystoides* resources as well as artificial cultivation.

## 2 Materials and methods

### 2.1 Collection and processing of distribution data for *Isodon amethystoides*

**Figure 1:**
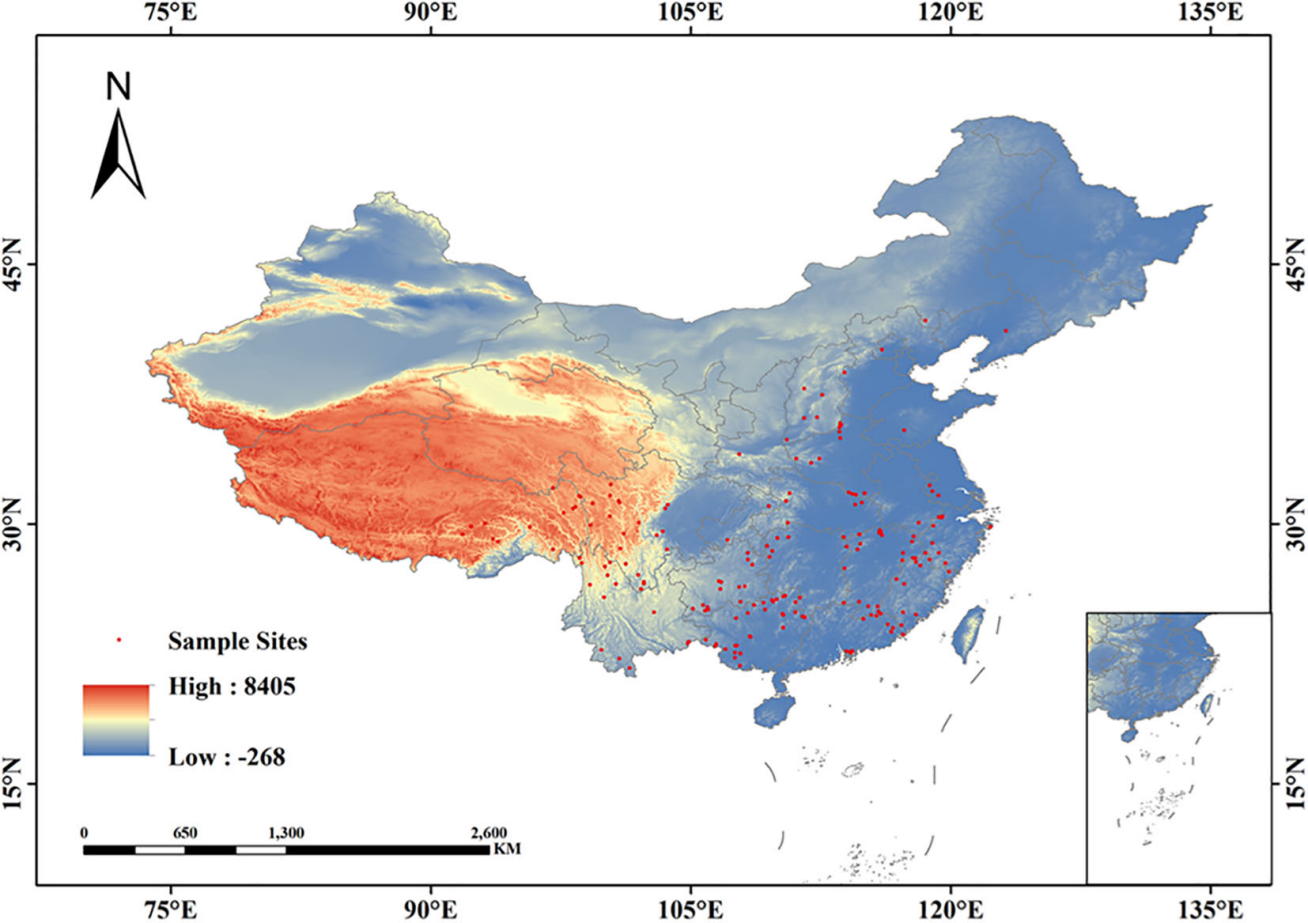
Spatial distribution of *Isodon amethystoides* occurrence points in China.

The distribution data of *Isodon amethystoides* were derived from the National Specimen Information Infrastructure (NSII, http://www.nsii.org.cn/), the Chinese Virtual Herbarium (CVH, https://www.cvh.ac.cn/), and relevant research literatures.This study only collected distribution sites of *Isodon amethystoides* after 1960. Distribution sites with clear latitude and longitude records were verified using Google Maps, and some distribution data with missing geographic coordinate information were supplemented. A total of 207 coordinate sites of *Isodon amethystoides* were collected from the Chinese Virtual Herbarium (CVH). To reduce spatial autocorrelation, a 5 km×5 km grid standard was adopted (Rodríguez-Castañeda et al., 2012; Zhang et al., 2019; Ji et al., 2021). Using ENMTools software, duplicate or geographically close distribution points were removed, resulting in 194 valid distribution points. The specific distribution locations. are shown in **Figure 1**.

### 2.2 Sources and processing of environmental data

In the environmental data used in this study, modern and future climate data were sourced from the WorldClim database (https://worldclim.org/), including 19 bioclimatic variables (Bio1–Bio19), precipitation from January to December, maximum temperature from January to December, minimum temperature from January to December, and average temperature from January to December. Three topographic factors (elevation, slope, and aspect) were extracted from DEM data. Soil texture type data were sourced from the Harmonized World Soil Database (HWSD). Future climate data were derived from CMIP6 (Coupled Model Intercomparison Project Phase 6), using the climate model of the Beijing Climate Center Climate System Model (BCC-CSM 2-MR). (Gao et al.). In the study, the 2030s (average value from 2021 to 2040), 2050s (average value from 2041 to 2060), 2070s (average value from 2061 to 2080), and 2090s (average value from 2081 to 2100) were used. Under the two extreme emission scenarios of SSP1-2.6 and SSP5-8.5 within the Shared Socioeconomic Pathways (SSPs), the spatial resolution of 90 environmental factors was standardized to 2.5 arc-minutes using bioclimatic data as the criterion. The specific information of the 90 environmental factors is shown in **Table 1**.

**Table 1:** Detailed information on the 90 environmental variables.

Variable code	Environmental factor	Variable code	Environmental factor
prec 1-12	January to December precipitation	alt	Altitude
tavg 1-12	January to December average temperature	slope	Slope
tmax 1-12	January to December maximum temperature	aspect	Aspect
tmin 1-12	January to December minimum temperature	zbyl	Vegetation Classification
bio1	Annual Mean Temperature	coarse	Coarse fragments
bio2	Mean Diurnal Range	sand	Sand
bio3	Isothermality	slit	Slit
bio4	Temperature Seasonality	clay	Clay
bio5	Max Temperature of Warmest Month	bulk	Bulk Density
bio6	Min Temperature of Coldest Month	ref_bulk	Reference Bulk Density
bio7	Temperature Annual Range	org_cbn	Organic Carbon Content
bio8	Mean Temperature of Wettest Quarter	ph	pH in water
bio9	Mean Temperature of Driest Quarter	n	Total nitrogen content
bio10	Mean Temperature of Warmest Quarter	cn	Carbon/Nitrogen ratio (C/N)
bio11	Mean Temperature of Coldest Quarter	cec_soil	CEC soil
bio12	Annual Precipitation	cec_clay	CEC clay
bio13	Precipitation of Wettest Month	teb	TEB
bio14	Precipitation of Driest Month	bsat	Base Saturation
bio15	Precipitation Seasonality	alum_sat	Aluminium saturation
bio16	Precipitation of Wettest Quarter	esp	Exchangeable Sodium Percentage
bio17	Precipitation of Driest Quarter	eq	Calcium Carbonate
bio18	Precipitation of Warmest Quarter	gypsum	Gypsum content
bio19	Precipitation of Coldest Quarter	elec_con	Electric Conductivity

Due to the potential high correlation among environmental variables that may affect modeling performance, key environmental variables were screened from the 90 variables for MaxEnt modeling through the following steps (Marshall et al., 2018). The 90 environmental variables and 194 distribution sites were input into MaxEnt to calculate the contribution rate of each variable to the model. Subsequently, the 90 variables were imported into ENMTools to compute the Pearson correlation coefficients (|r|) between any two variables; Variables with |Pearson’s r| < 0.8 were selected, while also retaining environmental factors with a contribution rate ≥ 0.5 in the initial MaxEnt model (Pascoe et al., 2019). Based on the above criteria, 11 environmental variables were finally retained for subsequent model establishment, optimization, and evaluation, namely bio_3, bio_4, prec_09, slope, prec_04, alt, eq, bsat, cec, coarse, and aspect.

### 2.3 Establishment, optimization, and evaluation of MaxEnt model

The processed distribution data of *Isodon amethystoides* were saved in CSV format and imported into MaxEnt together with the screened environmental factors. The model parameters were set as follows: 25% of the distribution data served as the test set (random test percentage), 75% as the training set, using the Bootstrap method with a default maximum of 10,000 background points. The random seed was checked to ensure model reproducibility, with the number of replicates set to 10, and the output format was logistic (Fourcade et al., 2014). In addition, the prediction results of the MaxEnt model are related to the regularization multiplier (RM), feature combination (FC), and the maximum number of background points. This study performed parameter optimization for the MaxEnt model using the Kuenm package in R4.4.1. Through systematic testing of 31 combinations of five feature classes (L/Q/H/P/T) and eight regularization multiplier gradients ranging from 0.1 to 4 (in increments of 0.5), we evaluated 248 parameter combinations (31×8) based on three criteria: ROC-AUC values, omission rates, and AICc values. The optimization process utilized 75% of the data for training, ultimately identifying the optimal model configuration while ensuring methodological rigor and significantly improving model performance. AUC (area under curve) refers to the area under the ROC (receiver operating characteristic) curve, which is typically used to test the accuracy of a model and is unaffected by the proportion of subjects in the analysis sample. The AUC value ranges from 0 to 1, where a higher value indicates better model fitting, higher construction accuracy, and greater credibility. An AUC value of 0.5–0.6 indicates model construction failure, 0.6–0.7 indicates poor simulation performance, 0.7–0.8 indicates general simulation performance, 0.8–0.9 indicates good simulation performance, and 0.9–1 indicates excellent simulation performance (Wang et al., 2020). In this study, the magnitude of AUC values was used to evaluate the predictive performance of each model. A larger AUC value indicates a stronger correlation between the modeled geographic distribution of *Isodon amethystoides* and environmental factors, suggesting better predictive performance of the model.

### 2.4 Data processing for MaxEnt modeling

To further investigate changes in the suitable habitat area of *Isodon amethystoides* under current and future scenarios, ArcGIS 10.8.1 software was used to classify and visualize its suitable habitats. The maximum test sensitivity plus specificity (MTSPS) threshold was selected for habitat classification because it integrates the sensitivity and specificity of the model and is directly calculated by MaxEnt. The ASCII format files from the average results of the MaxEnt model were imported into ArcGIS. Using the Reclassify tool, the habitat suitability was categorized into four classes: unsuitable (0–MTSPS), low suitability (MTSPS–0.5), moderate suitability (0.5–0.7), and high suitability (0.7–1). The distribution areas of these suitability classes were then calculated based on the number of raster cells in each class using ArcGIS.

In ArcGIS, the current and future suitable habitats of *Isodon amethystoides* were dichotomized into unsuitable (0–MTSPS) and suitable (MTSPS–1) areas. By comparing the current and future distributions, the habitat changes were classified into three categories: expansion areas, contraction areas, and stable areas. The geometric centroid of the suitable habitat for *Isodon amethystoides* serves as the distribution center point of the suitable habitat, and the position of this center point represents the overall spatial location of its suitable habitat. Under the assumption that *Isodon amethystoides* has migration ability and ignoring natural factors such as interspecific interactions, the centroid of the suitable habitat for *Isodon amethystoides* under different climatic scenarios and periods was calculated using the Zonal Geometric Statistics tool in ArcGIS. Vector files depicting the direction and magnitude of centroid changes between adjacent periods were generated to illustrate migration trends and distances.

## 3 Results

### 3.1 Model optimization and accuracy evaluation

**Figure 2:**
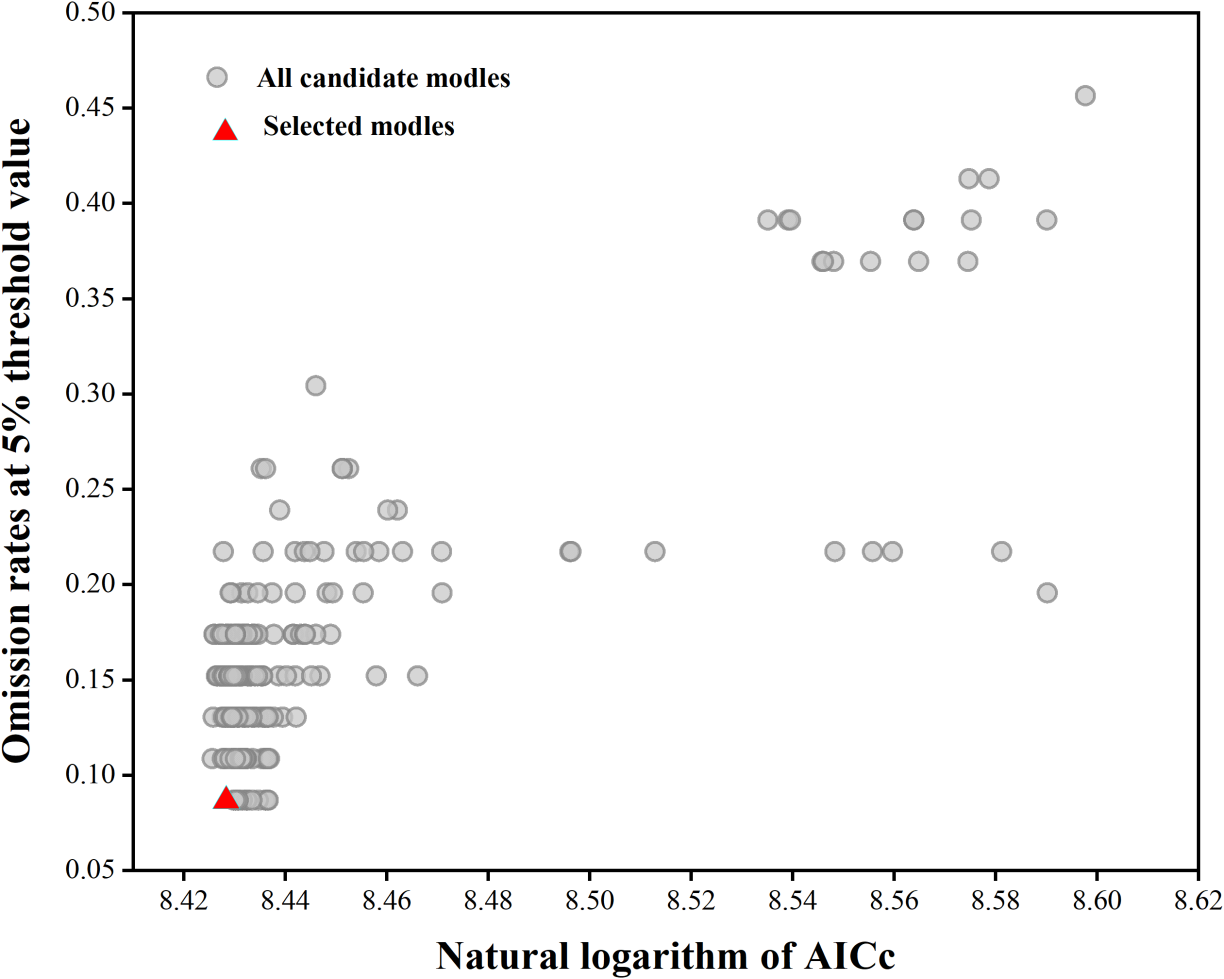
MaxEnt model parameter optimization results.

In the Maxent model, the Mean AUC Ratio represents the predictive ability of the model relative to random predictions, with higher values indicating better predictive performance. When using the default parameters in Maxent (FC = LQPH, RM = 1), the Mean AUC Ratio was 1.660. Parameter optimization was performed using the Kuenm package, selecting parameters FC = TH and RM = 3.5. Under these settings, the Mean AUC Ratio was 1.5642, significantly higher than the result obtained with default parameters (FC = LQPH, RM = 1, Mean AUC Ratio = 1.660). Additionally, the optimized model exhibited a lower AICc value, where a lower AICc indicates better model fit and lower complexity (**Figure 2**), suggesting the optimized model has superior interpretability. Therefore, this study selected FC = TH and RM = 3.5 as the parameter conditions for establishing and predicting the final distribution model of *Isodon amethystoides*. After running the initial and final models, it was found that under the optimized parameter conditions, the AUC value of the MaxEnt model decreased from 0.959 in the initial model to 0.902. The moderate decrease in AUC value indicates that the model is now more stable, avoiding overfitting and potentially leading to more reliable predictions.

### 3.2 Influence of key environmental variables on distribution of *Isodon amethystoides*

**Figure 3:**
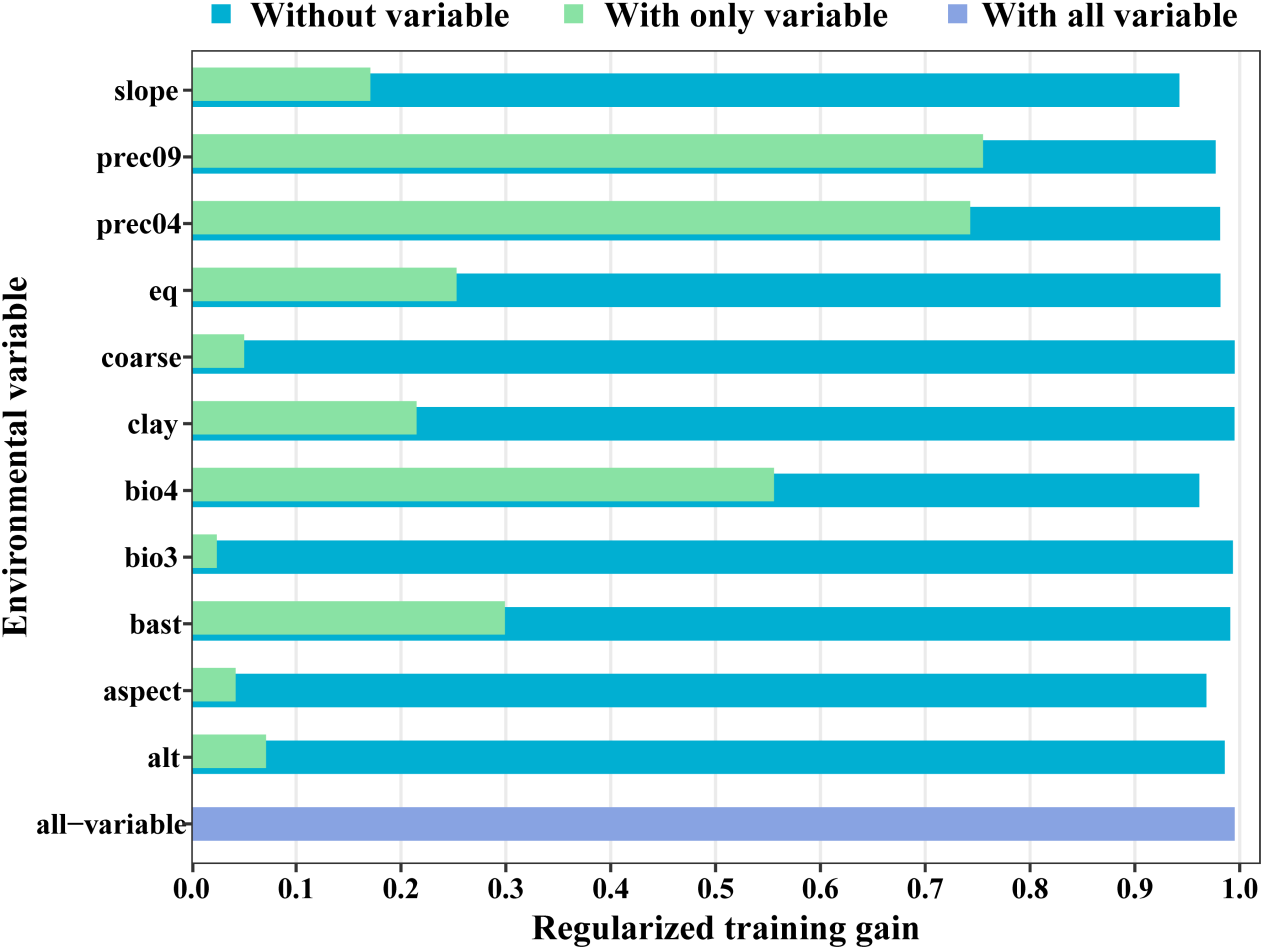
Results of jackknife test for the importance of the variables for MaxEnt.

**Figure 4:**
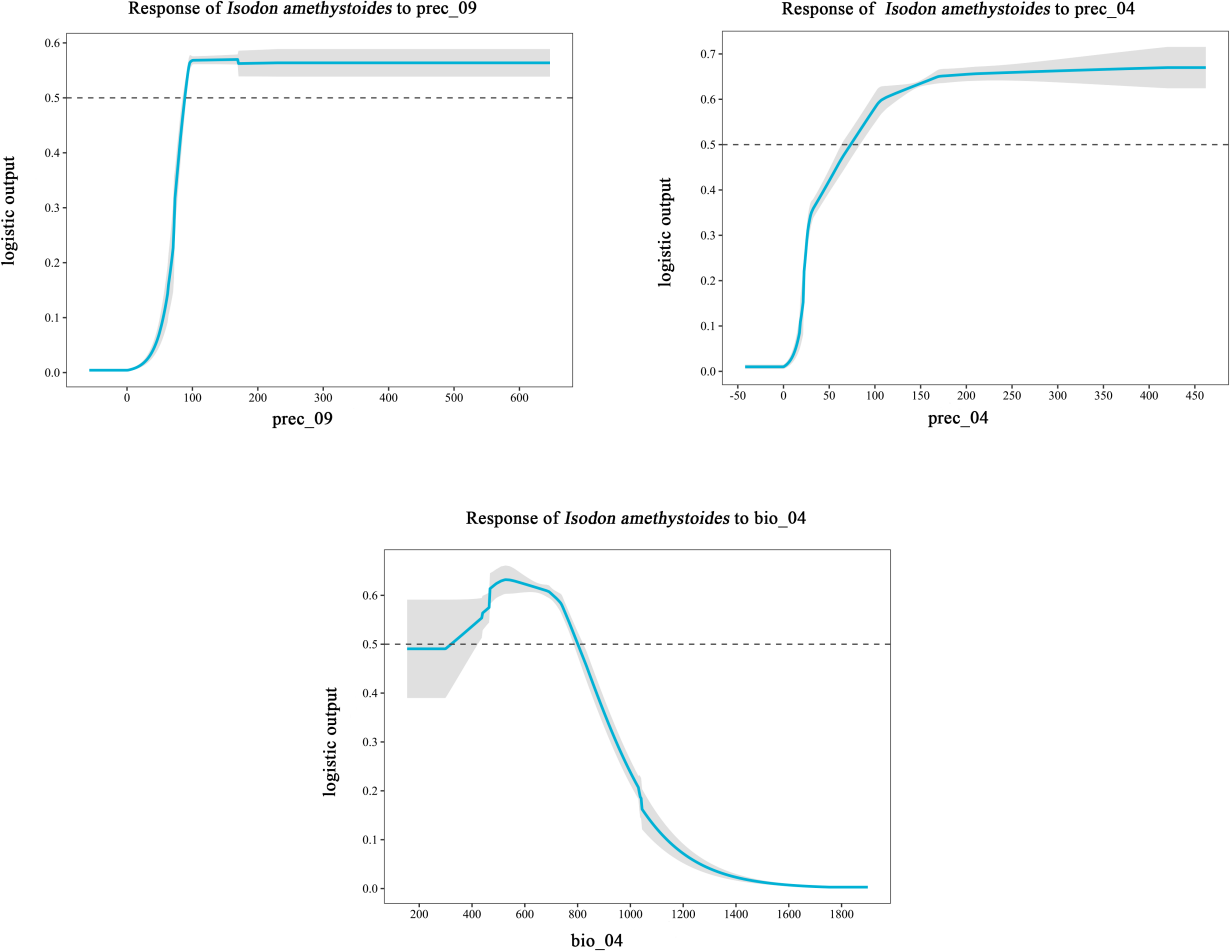
Response curves of key environmental variables (bio_04, prec_04, prec_09) influencing the suitable distribution of Isodon amethystoides.

The MaxEnt model was used to analyze the impact of 11 major environmental variables on the distribution of *Isodon amethystoides*. According to the contribution rates, prec_09 and prec_04 were the primary factors in model construction, with a cumulative contribution rate of 69.6%. Among them, prec_09 alone accounted for 46.9% of the contribution. The environmental variables with minor impacts included bio_04 (13.3%), slope (7.3%), alt (1.4%), eq (4%), bsat (1%), aspect (3%), cec-clay (0.1%), and bio_3 (0.2%). Meanwhile, the importance of each environmental variable was analyzed using the Jackknife method based on the generated results. According to the Jackknife test, when running the model with a single environmental variable, bio_4, prec_09, and prec_04 exhibited the highest Regularized Training Gain values. Therefore, bio_4, prec_09, and prec_04 were identified as the main environmental variables affecting the suitable distribution of *Isodon amethystoides*. (**Figure 3**) Response curves were plotted for key environmental variables (**Table 2**). When the logical output value exceeds 0.5, the corresponding environmental conditions are more suitable for the survival of this plant. Therefore, the most suitable conditions for the survival of *Isodon amethystoides* occur when the standard deviation of temperature seasonality ranges from 321.61 to 799.85, April precipitation ranges from 73.92 to 434 mm, and September precipitation ranges from 88.81 to 588 mm (**Figure 4**).

**Table 2:** The contribution rate of environmental variables.

Variable code	Environmental factor	Unit	Percent contribution/%	Permutation importance/%
prec_09	Precipitation in September	mm	46.8%	46.8%
prec_04	Precipitation in April	mm	22.8%	14.8%
bio_4	Temperature Seasonality		13.3%	23.3%
slope	Slope	degree	7.3%	10.6%
eq	Calcium Carbonate	%	4%	6.2%
aspect	Aspect	rad	3%	6.7%
alt	Altitude	m	1.4%	4.7%
bsat	Base Saturation	%	1%	2.9%
bio_3	Isothermal property	%	0.2%	0.7%
clay	Viscosity Content	%	0.1%	0.3%
coarse	Fineness of soil sand Particles	mm	0%	0%

### 3.3 Suitable distribution of *Isodon amethystoides* under current climatic conditions

**Figure 5:**
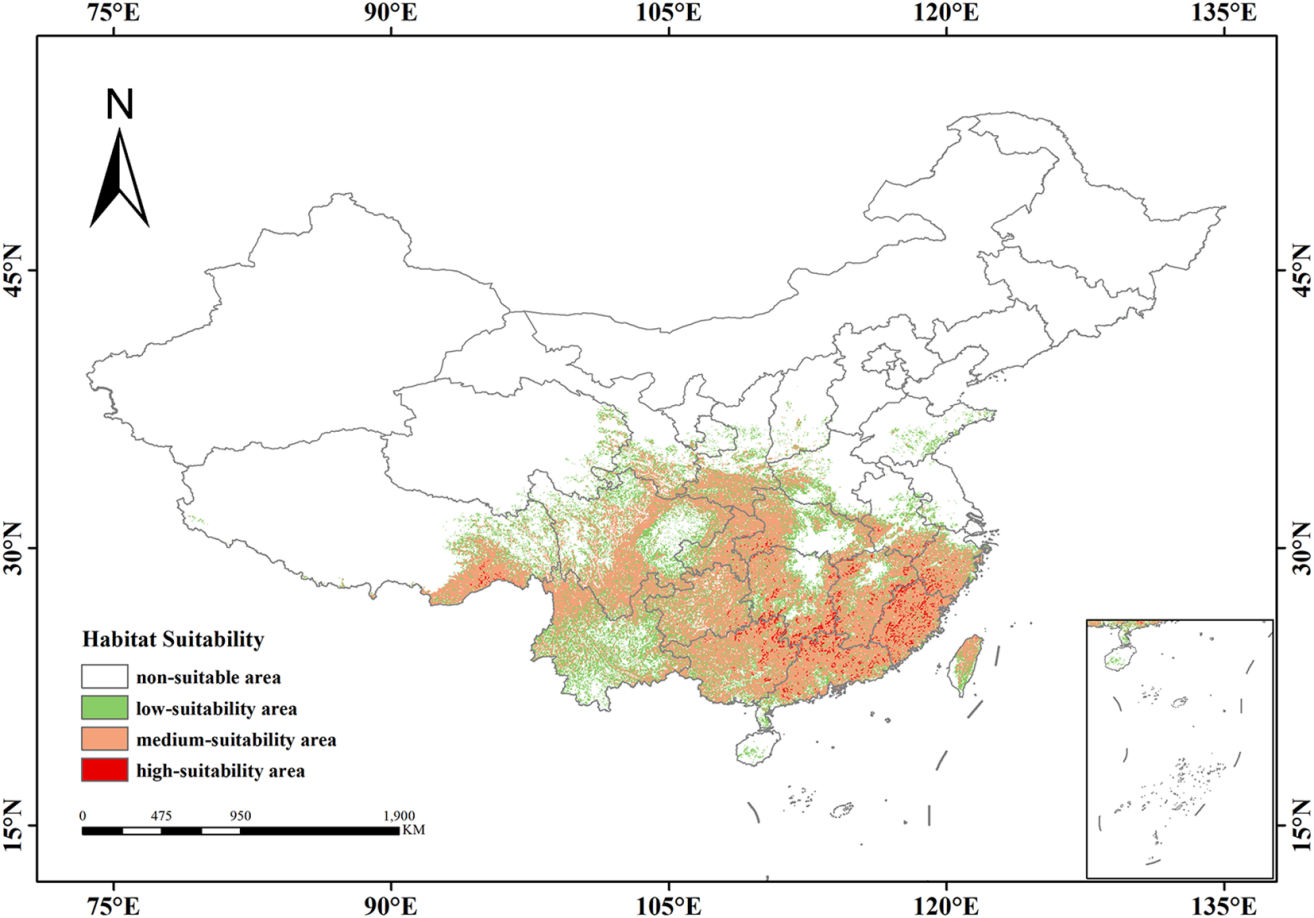
Potential suitable distribution of *Isodon amethystoides* under current climatic conditions.

Under current climatic conditions, the total suitable habitat area of *Isodon amethystoides* in China is 2.08×10^6^ km², accounting for 21.66% of China’s total land area. It is mainly distributed in Central China, East China, and South China. The high-suitability areas are mainly concentrated in the southeastern coastal regions, with an area of 5.45×10^4^ km², accounting for 0.56% of China’s total land area. These areas include parts of South China (e.g., Guangdong, Guangxi) and East China (e.g., Fujian, Jiangxi), forming relatively concentrated distribution patches with high ecological suitability. The moderate-suitability areas have a wider distribution, covering large parts of Central China such as Hubei and Hunan. The transitional distribution areas include parts of South China (e.g., Guangxi, Guangdong) and Southwest China (e.g., Sichuan, Chongqing), with a total area of 1.329×10^5^ km², accounting for 13.8% of China’s land area. Serving as ecological corridors connecting low-suitability and high-suitability zones, these areas form extensive continuous or semi-continuous distribution belts on the map. The low-suitability areas are mainly distributed in Southwest, North and East China, with an area of 6.99×10^4^ km², accounting for 7.27% of China’s total land area. They are in a relatively scattered distribution, existing in the form of spots and interspersed with other suitability grade areas (**Figure 5**).

### 3.4 Suitable distribution of *Isodon amethystoides* under future climate conditions

**Figure 6:**
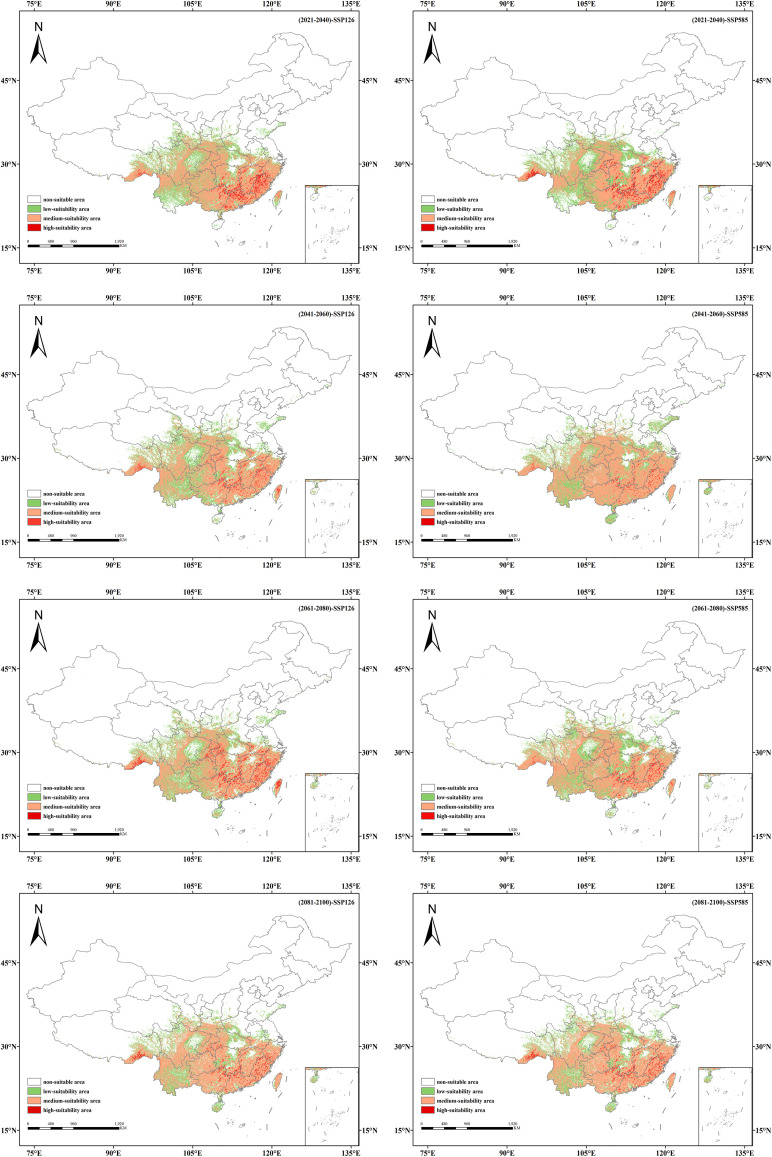
Suitable habitat distribution of *Isodon amethystoides* under different future climate scenarios.

Based on MaxEnt predictions for two emission scenarios corresponding to four future periods (2021–2040, 2041–2060, 2061–2080, 2081–2100), the future suitable habitat distribution of *Isodon amethystoides* in China and the areas of different suitability grades divided by MTSPS were obtained (**Table 3**, **Figure 6**).

**Table 3:** Area of suitable habitats for *Isodon amethystoides* under current and future climate scenarios by suitability levels.

Decade scenarios	Predicted area (× 10^4^ km^2^)				
	Low habitat suitability	Medium habitat suitability	High habitat suitability	Unsuitable habitat	Total suitable area
Current	69.96	132.93	5.45	753.99	208.35
2030s-SSP1-2.6	70.66	148.32	9.98	733.38	228.96
2050s-SSP1-2.6	76.60	145.94	9.68	730.12	232.23
2070s-SSP1-2.6	68.27	147.97	11.36	734.74	227.61
2090s-SSP1-2.6	66.38	165.66	6.44	723.86	238.48
2030s-SSP5-8.5	70.53	138.38	12.15	741.28	221.069
2050s-SSP5-8.5	63.62	180.78	3.20	714.74	247.61
2070s-SSP5-8.5	69.68	156.91	6.14	729.62	232.73
2090s-SSP5-8.5	56.59	153.37	7.38	744.99	217.36

Under the SSP1-2.6 scenario, the total suitable habitat area of *Isodon amethystoides* shows an overall increasing trend compared to the current climate. By the 2090s, the total suitable habitat area is expected to increase by 3.02×10^5^ km², among which the area of moderate-suitability areas has been continuously increasing, with an estimated increase of 3.27×10^5^ km². However, the area of low-suitability areas has been showing a decreasing trend, reaching 3.58×10^4^ km² by the 2090s. Under the SSP5-8.5 scenario, the total suitable habitat area of *Isodon amethystoides* exhibits a trend of initial increase followed by a decrease, which may be attributed to future climate precipitation given that rainfall is a primary factor affecting the species’ growth. In the 2050s, the total suitable habitat area will reach a peak of 2.47×10^6^ km², representing an increase of 3.92×10^5^ km² compared to the current climate. However, the area of high-suitability habitats shows an overall decreasing trend.

### 3.5 Spatial pattern changes in the future potential distribution of *Isodon amethystoides*

**Figure 7:**
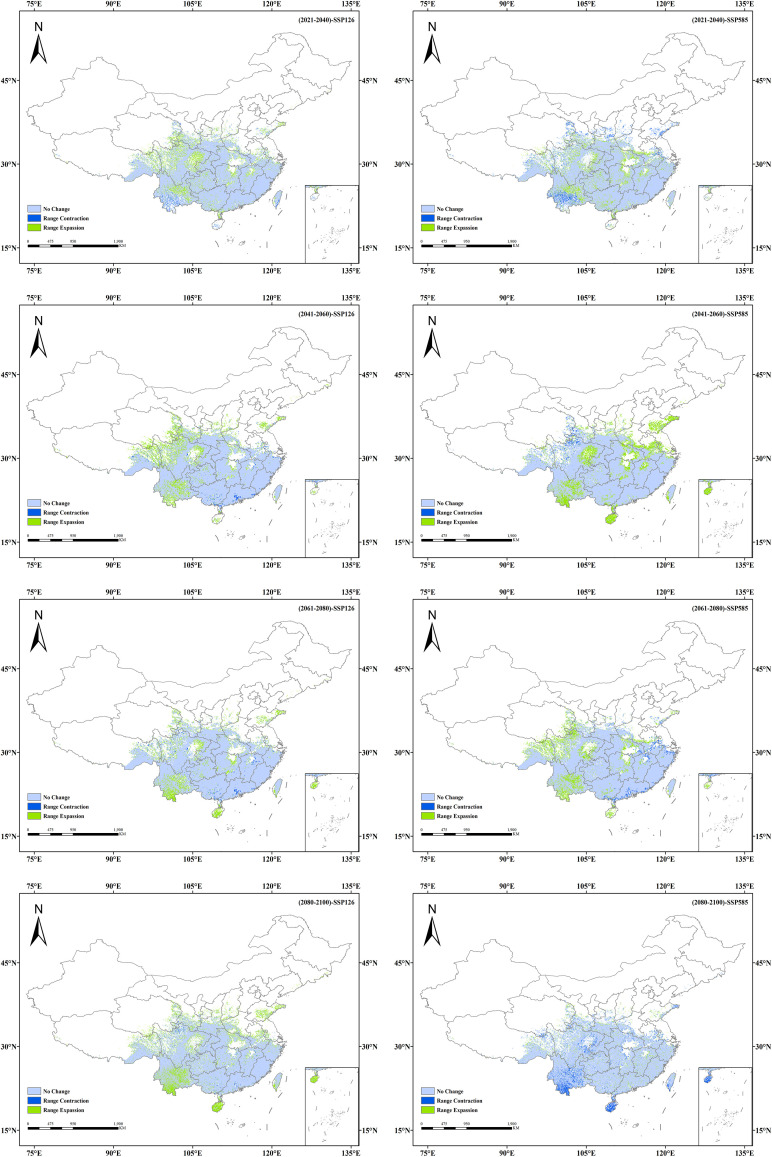
Spatio-temporal changes in potential suitable habitats of *Isodon amethystoides* under future climate scenarios.

The suitable habitat distributions of *Isodon amethystoides* under eight future climate scenarios were compared with those under the current scenario (**Figures 6**, **7**). It can be seen that under the SSP1-2.6 scenario, the suitable habitat of *Isodon amethystoides* exhibits the characteristics of northward expansion and southward contraction. The low-suitability zones show minor fluctuations in partial areas in the early stage, but generally tend to shrink in the later stage. Especially in the central and southern regions, the green coverage (low-suitability zones) gradually decreases, and many low-suitability areas transition to moderate and high-suitability zones, particularly in the Sichuan Basin and the margins of the Yunnan-Guizhou Plateau where low-suitability zones are more concentrated. The moderate-suitability zones show relative changes, with certain expansion in parts of northern regions in the later stage. For example, in South China and the Jiangnan Hills, the moderate-suitability zones were initially sparse, expanded to North China and Northeast China in the mid-term, and significantly increased in Central China, the Yangtze River Delta, and the Pearl River Delta in the later stage. Due to the expansion of low-suitability zones and the reduction of high-suitability zones, the moderate-suitability zones gradually expand. In particular, during the 2080s, some low-suitability areas may be upgraded to moderate-suitability areas, indicating a gradual improvement in environmental conditions. In addition, the high-suitability areas first show an expansion trend, rapidly expanding in the southeastern coastal areas in the early stage, stabilizing in the southern coastal areas in the middle and late stages, and slowing down or even locally contracting in the late stage. Some original high-suitability areas have transitioned to moderate and low-suitability areas, but core regions still remain. And the transformation of suitable habitats in different periods is closely related to regional climate and topographic effects. In summary, the low-suitability areas gradually decrease in the later stage, the moderate-suitability areas show little overall change, and the high-suitability areas first increase and then locally decrease, which all reflect the impact of environmental changes on plants.

Under the SSP5-8.5 scenario, the low-suitability areas show a contracting trend in some regions during the early stage, especially in provinces such as Hunan, Guizhou, and Yunnan. In the middle and late stages, they expand in Shandong Province as well as the Bohai and Yellow Sea areas, indicating an increase in the proportion of low-suitability regions and an overall shift of suitable habitats toward lower suitability. And the moderate-suitability areas show an interwoven trend of expansion and contraction, with the orange zones (moderate-suitability areas) tending to shrink in some regions. They expand rapidly in the early stage, covering the southeastern coast, East China, and Southwest China, but the orange coverage decreases in parts of these areas in the later stage, indicating reduced stability of moderate-suitable habitats. The high-suitability areas expand in the early stage in the southeastern coastal regions, Guangdong-Guangxi areas, southeastern Tibet, and southern Hunan, but show a contracting trend in the middle and late stages. However, the overall area of suitable habitats remains relatively stable.

### 3.6 Centroid migration of suitable habitats in future periods

**Figure 8:**
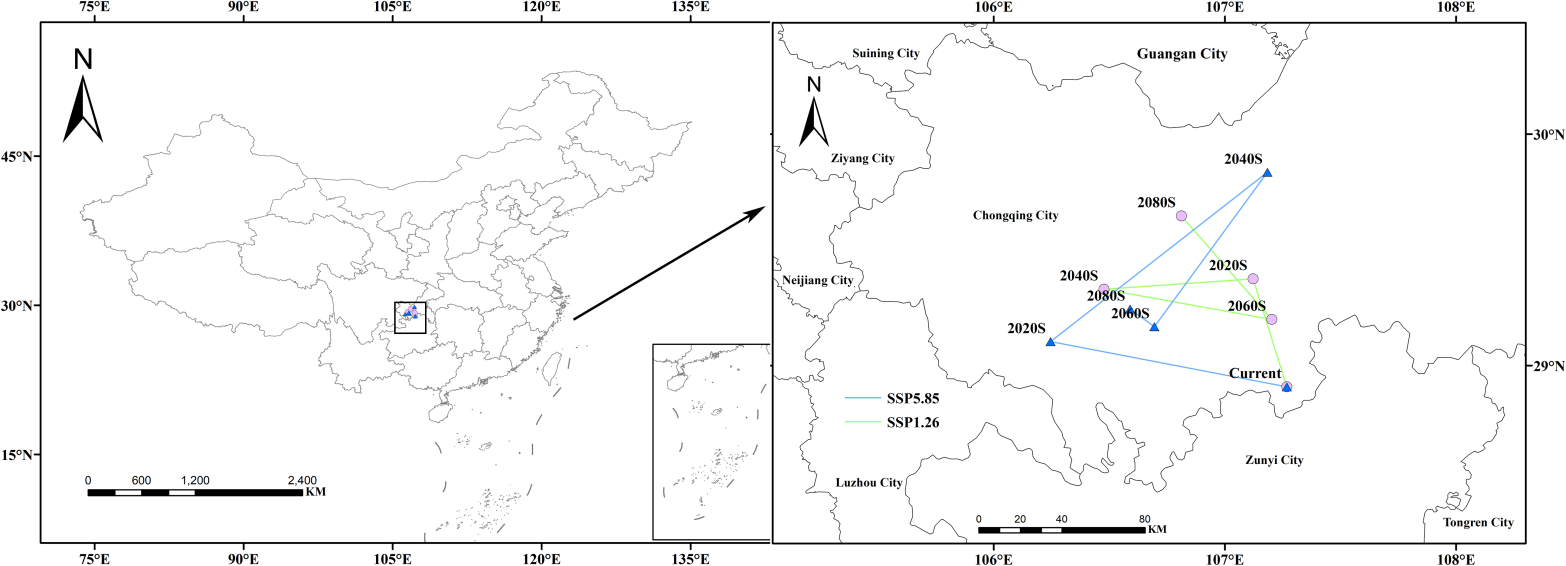
Migration trajectory of the geometric centroid of suitable areas for *Isodon amethystoides* under future climate scenarios.

Using the MTSPS value (MTSPS = 0.4012) as the threshold, *Isodon amethystoides* suitable habitats were divided into suitable and unsuitable areas. Based on the temporal shifts in the centroid of suitable habitats for *Isodon amethystoides*, we mapped the migration trajectory of the distribution centroid (**Figure 8**). Under the current climate, the centroid of suitable habitats for *Isodon amethystoides* is located in Nanchuan District, Chongqing (107.10°E, 29.16°N). Under the SSP1-2.6 emission scenario, from 2021 to 2040, the centroid migrates 54.99 km northwest to the boundary between Fuling District and Nanchuan District, Chongqing (107.22°E, 29.69°N); Under the SSP1-2.6 emission scenario, the centroid of its suitable habitat migrated 54.99 km northwest to the boundary between Fuling District and Nanchuan District of Chongqing (107.22°E, 29.69°N) during 2021–2040; During 2041–2060 under the SSP1-2.6 emission scenario, the centroid further migrated 63.08 km northwest to Banan District, Chongqing (106.48°E, 29.41°N); During 2061–2080, the centroid migrated 73.00 km southeast to the central part of Nanchuan District, Chongqing (107.10°E, 29.17°N); while during 2081–2100, it moved 32.82 km northwest to Jiangbei District, Chongqing (106.57°E, 29.61°N); Under the SSP5-8.5 emission scenario, the centroid of its suitable habitat migrated 103.19 km northwest to Jiangjin District, Chongqing (106.26°E, 29.30°N) during 2021–2040; During 2041–2060, the centroid migrated 123.32 km northeast to Changshou District, Chongqing (107.08°E, 29.86°N); During 2061–2080, the centroid migrated 88.81 km southwest to the northern part of Qijiang District, Chongqing (106.60°E, 29.35°N); During 2081–2100, the centroid migrated 13.44 km northwest to Banan District, Chongqing (106.54°E, 29.41°N).

## 4 Discussion

The Maxent model infers species requirements based on specific algorithms to predict their potential distribution, and it can be applied to ecological conservation. However, it cannot predict or solve all problems (Fernandes et al., 2018; Zhen et al., 2018). The AUC value of the optimized model is 0.902, indicating that the prediction of this model has high reliability (Elith et al., 2011). Experimental results show that the growth of *Isodon amethystoides* is primarily related to precipitation and temperature. Among these, precipitation in September has the greatest impact on its growth, with a contribution rate of 46.8%. This is followed by precipitation in April and the standard deviation of temperature seasonality, indicating that the distribution of *Isodon amethystoides* is mainly determined by these three environmental factors (Root et al., 2003).

Currently, *Isodon amethystoides* in China is mainly produced in areas from north of the Qinling-Huaihe Line to North China and southern Northeast China, as well as southeastern Tibet. Experimental results also confirm that under the current climate scenario, *Isodon amethystoides* is mainly distributed in Central China, East China, South China and other regions. The total suitable habitat area in China is 2.08×10^6^ km², accounting for 21.6% of China’s total land area. The experimental model’s prediction of the potential suitable habitat for *Isodon amethystoides* highly coincides with the actual distribution. Under future climate change scenarios, the area of potential suitable habitats for *Isodon amethystoides* generally shows an increasing trend, but with fluctuating characteristics by year. This volatility may be related to future changes in precipitation. Especially in some extremely dry years, the decrease in precipitation leads to the reduction of local suitable habitat area (Liu et al., 2005). Due to the impact of future climate change, the centroid of *Isodon amethystoides* suitable habitats shifts to high-latitude areas, specifically manifested as the centroid of suitable regions expanding northwestward. This indicates that *Isodon amethystoides* migrates to new habitats to adapt to the new natural environment (Lamprecht et al., 2018; Abdelaal et al., 2019). In addition, against the backdrop of global warming, precipitation and temperature are increasing year by year, which is more conducive to the distribution of *Isodon amethystoides* and will also expand its plantable range (Casseau et al., 2015). Furthermore, the centroid shift reflects an active climate adaptation strategy, wherein the species tracks optimal hydrothermal conditions to maintain ecological competitiveness. Notably, unlike the predominant altitudinal shift observed in most species, *Isodon amethystoides* exhibits a latitude-priority adaptation strategy—expanding toward higher latitudes while retaining its core elevational range (800–1200 m). This unique migration pattern creates newly suitable habitats for potential cultivation expansion. In addition, the existing production areas in Guizhou need to be maintained in the short term while improving soil acidity, whereas the core cultivation areas should be gradually relocated to central Yunnan in the long term, as this region shows higher habitat suitability matching under the SSP1-2.6 scenario.

Although the model’s prediction accuracy is high (AUC value reaches 0.902), its results still belong to the category of theoretical deduction, and the actual suitable habitats may be affected by more complex factors. For instance, factors such as the process of economic development, changes in land use patterns, government policy orientation, and human activity interference may all have non-negligible effects on the actual distribution of *Isodon amethystoides* (Xu et al., 2018; Aneva et al., 2020; Wang et al., 2022). Therefore, follow-up studies can incorporate multiple types of environmental variables into ecological suitability modeling, which can enhance the authenticity and accuracy of the model (Zandalinas et al., 2021).

## 5 Conclusion

This study, based on the optimized MaxEnt model, theoretically predicts the suitable distribution of *Isodon amethystoides* in China under future climate change. The results show that the growth of *Isodon amethystoides* is mainly affected by the standard deviation of temperature seasonal variation and precipitation. When the logistic output value is greater than 0.5, the corresponding environmental factor values are conducive to plant growth. The standard deviation of temperature seasonal variation within the range of 321.61–799.85°C, April precipitation within 73.92–434 mm, and September precipitation within 88.81–588 mm are most suitable for the survival of *Isodon amethystoides*. Under current climatic conditions, the suitable habitats for *Isodon amethystoides* are mainly distributed in Central China, East China, and South China, with a total area of 2.08×10^6^ km², accounting for 21.6% of China’s total land area. Under future climate change scenarios (SSP1-2.6 and SSP5-8.5), the total area of suitable habitats generally shows an increasing trend. On the other hand, the geometric centroid of the suitable habitat will show a trend of migrating northwestward. This indicates that key environmental factors such as temperature and precipitation may affect the geographical distribution pattern of *Isodon amethystoides*. On the other hand, the geometric centroid of the suitable habitat will show a trend of migrating northwestward.

## Data Availability

The datasets presented in this article are not readily available because the data is sourced from an open database, and the predicted results of the suitable areas generated by the model do not contain any sensitive information. Requests to access the datasets should be directed to (NSII, http://www.nsii.org.cn/).
